# Parallel Inhibition of Interleukin 4 (IL-4)/Interleukin 13 (IL-13) and Janus Kinase 1 (JAK1) in Atopic Dermatitis: Beyond the Traditional Biologic Paradigm

**DOI:** 10.7759/cureus.98589

**Published:** 2025-12-06

**Authors:** Ana G Perez-Romero, Martha A Rangel, Ana B Crocker Sandoval, Mariana Ruiz Leon, Martha Aceves

**Affiliations:** 1 Dermatology, Hospital Regional "Dr. Valentín Gómez Farías" Institute for Social Security and Services for State Workers (ISSSTE), Zapopan, MEX; 2 Dermatology, Hospital San Javier, Guadalajara, MEX

**Keywords:** abrocitinib, atopic dermatitis, combination drug therapy, dupilumab, jak1 inhibitors

## Abstract

Moderate-to-severe atopic dermatitis (AD) substantially impairs quality of life; beyond pruritus, cutaneous pain is clinically relevant. Although dupilumab and abrocitinib have transformed management, a proportion of patients fail to achieve sustained remission on monotherapy. In refractory cases, combining both agents may optimize control of pruritus and pain and enhance overall response. We present three severe AD cases successfully treated with dupilumab plus abrocitinib. All three patients exhibited favorable changes in clinimetric scores, with prompt pruritus relief and a clinically stable course during follow-up. Dupilumab reduces pruritus and flares and significantly improves quality of life, yet a relevant proportion exhibits incomplete response or relapse. In this setting, abrocitinib, a selective Janus kinase 1 (JAK1) inhibitor, produces a rapid effect on pruritus and pain, thus providing a dual effect with rapid, durable control of pruritus and pain along with a significant improvement in quality of life in refractory severe AD, supporting its use in carefully selected patients under close clinical and laboratory monitoring.

## Introduction

Atopic dermatitis (AD) is a chronic inflammatory dermatosis that frequently presents in moderate-to-severe forms with substantial impairment of quality of life. While pruritus remains the hallmark symptom, cutaneous pain is increasingly recognized as a clinically meaningful component, particularly in severe disease [1,2]. Targeted therapies, such as dupilumab, which inhibits interleukin 4 (IL-4)/interleukin 13 (IL-13) signaling via IL-4Rα blockade, and abrocitinib, a selective Janus kinase 1 (JAK1) inhibitor that modulates downstream signaling of multiple cytokines-have transformed care [1,3,4]. Nevertheless, a considerable proportion of patients fail to achieve durable clinical remission on monotherapy [2,5]. In refractory disease, concomitant use of both agents has emerged as a therapeutic alternative; this dual approach may enhance control of complex symptoms, including pruritus and pain, and improve overall treatment response in severe AD [2,6]. Herein, we present three cases of severe AD inadequately controlled with monotherapy and successfully managed with combined dupilumab and abrocitinib. The PROMs (Patient Reported Outcome Measures) and CROMs (Clinician Reported Outcome Measures) applied at baseline and at six months in each patient were EASI (Eczema Area and Severity Index) [7], POEM (Patient Oriented Eczema Measure) [8], worst pruritus NRS (numeric rating scale) [9], and worst pain NRS [10].

## Case presentation

Patient 1

**Figure 1:**
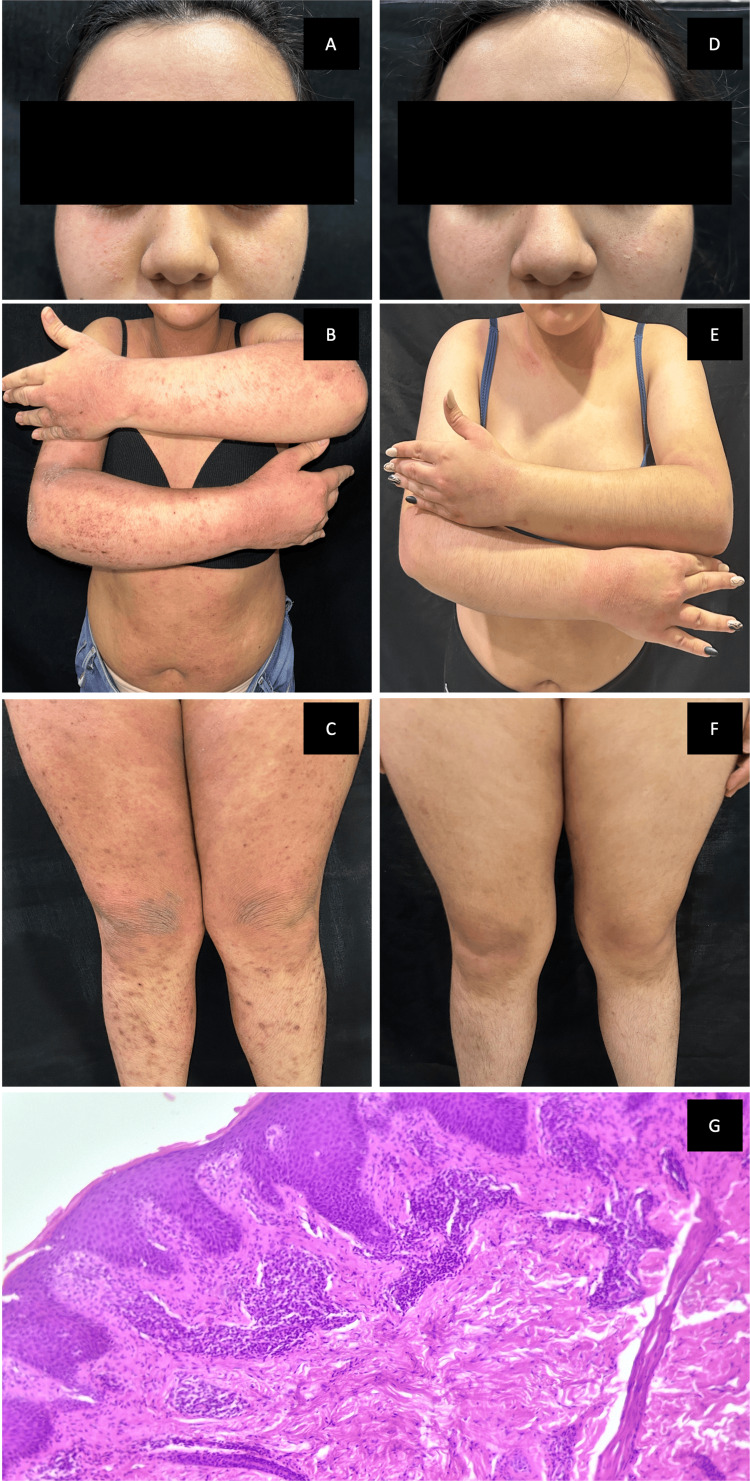
Case 1 (A-C) Baseline: generalized, symmetric dermatitis involving the face and extremities, characterized by diffuse erythema, excoriated papules, lichenified plaques, and xerosis. (D-F) Nine-month follow-up: marked clinical remission with resolution of erythema and active inflammation, leaving only residual postinflammatory hyperpigmented macules and mild lichenification. G) Pathology section showing mild irregular acanthosis, hyperkeratosis, spongiosis, dermal edema and perivascular lymphocytic infiltrate. For the minor, legal guardian consent and patient assent were obtained.

Patient 1 is a 16-year-old girl with a history of infantile eczema and diaper dermatitis that progressed to AD during the first year of life, with comorbid allergic rhinitis and conjunctivitis. She had received emollient-based skin care and multiple courses of high-potency topical and systemic corticosteroids without adequate disease control. Baseline clinimetric scores demonstrated severe active disease: EASI 43, pruritus NRS 10/10, pain NRS 9/10, POEM 22, serum IgE 13,629IU/mL (reference range: 0.0-378.0 IU/mL), and eosinophils 1.2×10^3/µL (reference range: 0-0.8x10^3/µL). Diagnosis of chronic eczematous dermatitis was confirmed by histopathology. Weight-tired dose dupilumab was initiated (65 kg), 300 mg subcutaneously every two weeks, in combination with cyclosporine 200 mg (3 mg/kg/day) once daily for two months, with an EASI 50 improvement during the first month. At month 2, however, she experienced a severe flare with persistent pruritus and pain. Cyclosporine was discontinued, and abrocitinib 100 mg once daily was started for two months (dose and duration limited by access) along with dupilumab. Thereafter, she achieved progressive pain reduction and sustained disease control over the ensuing nine months (Figure 1; Table 1).

**Table 1:** Clinical course of three patients with severe atopic dermatitis treated with combination therapy with dupilumab and abrocitinib Baseline scores and outcomes at six months are shown for EASI, pruritus NRS, pain NRS, and POEM. AD: atopic dermatitis; NRS: numeric rating scale; POEM: Patient Oriented Eczema Measure; EASI: Eczema Area and Severity Index.

	Patient 1	Patient 2	Patient 3
Age	16 years	39 years	32 years
Sex	F	F	M
AD onset	Infancy	Age 3 years	Age 2 years
Previous treatments	Topical and systemic corticosteroids	Topical and systemic corticosteroids; dupilumab for 12 months	Topical and systemic corticosteroids; dupilumab for 12 months
Combination treatment	Dupilumab + abrocitinib 100 mg (2 months)	Dupilumab + abrocitinib 200 mg (3 months)	Dupilumab + abrocitinib 200 mg (3 months)
Baseline clinimetry			
EASI	43	40	50
NRS pruritus	10	10	10
NRS pain	10	6	8
POEM	22	14	18
Clinimetry at six months follow-up			
EASI	8.3	6.6	7.1
NRS pruritus	2	1	2
NRS pain	0	0	0
POEM	4	2	3

Patient 2

**Figure 2:**
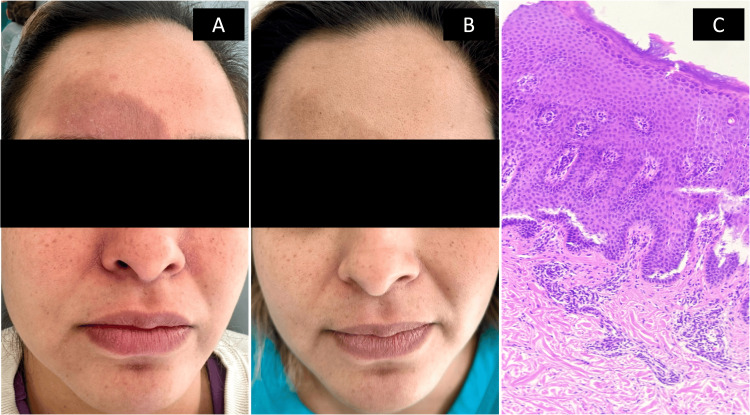
Case 2 A) Baseline assessment while on monotherapy: generalized dermatosis with lichenified erythematous plaques, papules, fissures, and excoriations; chronic course. B) Six-month assessment during dual therapy: disseminated, symmetric dermatosis characterized by residual postinflammatory hyperpigmented macules and a few mildly lichenified plaques. C) Pathology showing acanthosis with spongiosis, some microvesicles, exocytosis of inflammatory cells and perivascular lymphohistiocytic inflammatory infiltrate.

Patient 2 is a 39-year-old woman with AD since age three years, allergic rhinitis, and a nevus of Ota. She had received suboptimal skin-care measures and multiple courses of high-potency topical and systemic corticosteroids. She was treated with dupilumab for one year, initial loading dose 600 mg followed by 300 mg every two weeks, achieving only a partial response with frequent flares. On clinical reassessment, disease remained moderate to severe and uncontrolled despite biologic therapy (POEM 14; EASI 40; pruritus NRS 10/10; pain NRS 6/10; serum IgE 10,423IU/mL; eosinophils 1.5x10^3/µL). Histopathology confirmed chronic eczematous dermatitis. Given inadequate control, abrocitinib 200 mg once daily was added to ongoing dupilumab for three months, resulting in a favorable response from the first month and at follow-up (Figure 2).

Patient 3

**Figure 3:**
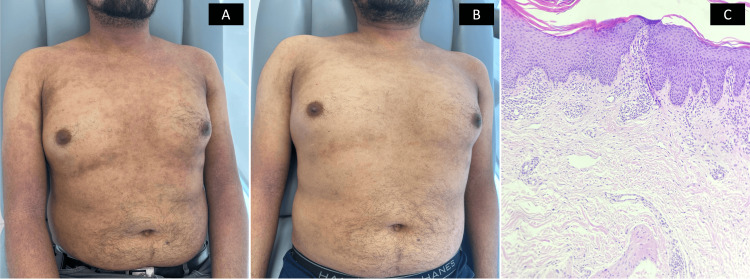
Case 3 A) Baseline assessment while on monotherapy: generalized, symmetric dermatosis with lichenified erythematous plaques, papules, fissures, and excoriations; chronic course. B) Six-month assessment during dual therapy: disseminated, symmetric dermatosis characterized by plaques with mild lichenification, some with erythema, and residual postinflammatory hyperpigmented macules; chronic course. C) The pathology shows irregular acanthosis, laminar hyperkeratosis, mild spongiosis and perivascular lymphohistiocytic inflammatory infiltrate.

A 32-year-old man with AD with disease onset at age two years was previously treated with topical calcineurin inhibitors and high-potency topical and systemic corticosteroids without sustained control. Dupilumab 600 mg loading dose followed by 300 mg every two weeks was initiated with an adequate initial response; however, after 12 months, he developed frequent flares. On reevaluation, he had EASI 50, pruritus NRS 10/10, pain NRS 6/10, POEM 18, serum IgE 11,714IU/mL, and eosinophils 1.2×10^3/µL, indicating severe impairment and persistent inflammatory activity. Histopathology confirmed chronic eczematous dermatitis. Given recalcitrant disease, abrocitinib 200 mg once daily was added while continuing dupilumab for three months, resulting in rapid improvement within weeks, progressive reductions in symptoms and clinimetric scores, and maintenance of a favorable response through six months of follow-up (Figure 3).

## Discussion

AD is a chronic inflammatory skin disease predominantly driven by type 2 immune responses; however, Th1, Th17, and Th22 axes also contribute to severe or advanced disease. Over recent decades, targeted therapies have transformed the management of moderate-to-severe AD, particularly with the introduction of dupilumab, a monoclonal antibody against IL-4 receptor alpha, the shared receptor subunit for IL-4 and IL-13. Dupilumab inhibits IL-4 signaling, which is central to IgE class switching, Th2 polarization, and eosinophil recruitment, while also blocking IL-13, a key mediator of barrier dysfunction, dermal fibrosis, and pruritus via peripheral neuronal pathways. Receptor blockade is associated with improvement in pruritus, fewer flares, and normalization of biomarkers such as IgE and TARC [1]. Nevertheless, 15%-38% of pediatric patients fail to achieve an adequate response after 16 weeks [2], and 35%-45% of adults experience relapse, partial response, or waning efficacy over time [3].

To address this unmet need, JAK inhibitors have emerged as therapeutic alternatives, including off-label use in combination regimens. Abrocitinib, an oral highly selective JAK1 inhibitor, interferes with intracellular signaling downstream of multiple cytokines, including IL-4, IL-13, IL-22, IL-31, IFN-γ, and TSLP [4]. In contrast to dupilumab, this mechanism provides broader pathway coverage; in addition to indirectly modulating IL-4 and IL-13, abrocitinib attenuates pruritogenic cytokines such as IL-31 and chronic inflammatory mediators such as IL-22, producing rapid reductions in pruritus and pain, likely related to modulation of IL-6 and pro-inflammatory neuropeptides involved in peripheral sensitization [5,6].

Accordingly, combining dupilumab and abrocitinib provides immunologic synergy. Dupilumab targets extracellular IL-4 and IL-13 signaling, whereas abrocitinib blocks a broader set of cytokines within the cell that drive inflammation, barrier dysfunction, and neurosensory sensitization. This approach has been documented in the literature, with treatment being well tolerated, few adverse effects such as conjunctivitis, and improvement of associated alopecia areata in one case [2]. 

Our findings are consistent with these data. In our three cases, combination therapy produced a marked decrease across clinimetric scales with approximately EASI75 improvement at six months, more than 50% reduction in pain within the first month, and complete pain resolution by six months, a response not achieved with monotherapy. We also observed substantial gains in quality of life at six months and no adverse events during follow-up. Overall, the dual approach yielded rapid improvement with favorable tolerability and facilitated attainment of treat-to-target goals [11].

Additional evidence supports these benefits. Song et al. reported that 82% of patients with severe AD inadequately controlled on dupilumab achieved a reduction of at least four points in pain scores after adding abrocitinib [6]. Hu et al. evaluated drug persistence in real-world settings and found that abrocitinib 200 mg/day achieved a 24-week persistence rate of 80% compared with 49% for dupilumab, with an even greater difference in patients with SCORAD >50, among whom persistence reached 93.75% [3].

From a practical standpoint, combination therapy with dupilumab and abrocitinib may be particularly useful in patients with severe disease, refractory symptoms such as chronic pain, or a history of dupilumab-associated adverse effects such as conjunctivitis, which appear less frequent when JAK inhibitors are used in combination [2]. Despite these benefits, potential risks should be considered. Although serious adverse events have been uncommon in reported cohorts, concomitant use of a biologic agent and a JAK inhibitor could increase the theoretical risk of infections, dyslipidemia, or hematologic abnormalities [2,4-6]. Consequently, this strategy should be reserved for refractory disease with close monitoring and predefined criteria to assess response, treatment duration, and safety.

## Conclusions

The combination of dupilumab and abrocitinib represents an innovative and promising therapeutic approach for refractory severe AD. Their complementary actions across multiple immunologic pathways translate into improvements in pruritus, pain, and quality of life. The available evidence, together with the results presented in this report, suggests a meaningful clinical benefit in patients who have not responded adequately to monotherapy. Nevertheless, controlled clinical trials and prospective studies are warranted to evaluate long-term safety, efficacy, and cost-effectiveness.
